# Cordycepin Reverses Cisplatin Resistance in Non-small Cell Lung Cancer by Activating AMPK and Inhibiting AKT Signaling Pathway

**DOI:** 10.3389/fcell.2020.609285

**Published:** 2021-01-15

**Authors:** Xiao-Zhong Liao, Ying Gao, Hong-Wei Zhao, Mi Zhou, Dan-Lei Chen, Lan-Ting Tao, Wei Guo, Ling-Ling Sun, Chu-Ying Gu, Han-Rui Chen, Zhi-Wei Xiao, Jia-Xing Zhang, Mei-Fang He, Li-Zhu Lin

**Affiliations:** ^1^Department of Oncology, The First Affiliated Hospital, Guangzhou University of Chinese Medicine, Guangzhou, China; ^2^Laboratory of General Surgery, The First Affiliated Hospital, Sun Yat-sen University, Guangzhou, China; ^3^Department of Oncology, The First Affiliated Hospital, Sun Yat-sen University, Guangzhou, China

**Keywords:** NSCLC, cisplatin, resistance, cordycepin, AMPK, AKT

## Abstract

Cisplatin (DDP) is the first-line chemotherapeutic agent against lung cancer. However, the therapeutic effect of DDP loses over time due to the acquired drug resistance in non-small cell lung cancer (NSCLC) cells. In recent years, the role of the traditional Chinese medicine (TCM) cordycepin (Cor) in cancer treatment has been attracting attention. However, the effects of Cor on DDP resistance in NSCLC are unclear. In the present study, we aimed to investigate the effects of Cor in combination with DDP on cell proliferation and apoptosis in NSCLC and explore possible underlying mechanisms. The cell proliferation and apoptosis were analyzed in NSCLC parental (A549) and DDP-resistant (A549DDP) cells treated with DDP alone or in combination with Cor both *in vitro* and *in vivo*. Different genes and signaling pathways were investigated between DDP-sensitive and DDP-resistant A549 cells by Gene Ontology (GO) and Kyoto Encyclopedia of Genes and Genomes (KEGG) analysis. The perturbations of the MAPK and PI3K-AKT signaling pathways were evaluated by Western blot analysis. Our data showed that Cor markedly enhanced DDP inhibition on cell proliferation and promotion of apoptosis compared to the DDP-alone group in both A549 and A549DDP cells. The synergic actions were associated with activation of AMPK; inhibition of AKT, mTOR, and downstream P709S6K; and S6 phosphorylation in the AKT pathway compared with DDP alone. Collectively, combination of Cor and DDP has a synergistic effect in inhibiting proliferation and promoting apoptosis of NSCLC cells in the presence or absence of DDP resistance. The antitumor activity is associated with activation of AMPK and inhibition of the AKT pathway to enhance DDP inhibition on NSCLC. Our results suggested that Cor in combination with DDP could be an additional therapeutic option for the treatment of DDP-resistant NSCLC.

## Introduction

Lung cancer is the most common cancer and the leading cause of cancer-related death worldwide. Non-small cell lung cancer (NSCLC) is the major (85%) histological subtype of the disease (Siegel et al., 2019). NSCLC is often diagnosed at an advanced stage in most patients. Cisplatin (DDP) is the first-line chemotherapeutic agent for the treatment of NSCLC owing to its effective anticancer activity. DDP reacts with DNA to induce its characteristic biological effects leading to DNA damage or activation of irreversible apoptotic program (Siddik, 2003). However, the antineoplastic effect of DDP is greatly hampered by drug resistance (Galluzzi et al., 2012). Most advanced NSCLC acquired DDP resistance leading to tumor growth or distant metastasis over time. Several molecular mechanisms of drug resistance have been explored including cancer heterogeneity, tumor microenvironment, and drug targets. One imperative mechanism is the alterations in drug metabolism by upregulating intracellular GSH level (Siddik, 2003). The increasing content of GSH is a major contributing factor to cisplatin resistance, because GSH can bind to drugs, interact with ROS, and participate in DNA repair processes (Traverso et al., 2013).

Cordycepin (3′-deoxyadenosine, Cor) is the main active compound of the traditional herbal medicine *Cordyceps militaris*, which has anti-inflammation, antiviral and antitumor effects (Panicali and Nair, 1978; Wu et al., 2007; Liao et al., 2015). A number of studies have presented that Cor has antitumor activities including induction of cell apoptosis, inhibition of cell proliferation, and migration (Koc et al., 1996; Lee et al., 2010). Moreover, Cor was revealed to be able to re-sensitize drug-resistant cancer cells in various tumors, including glioblastoma, ovarian cancer, and lung cancer (Wang et al., 2017; Bi et al., 2018; Cho and Kang, 2018; Wei et al., 2019). Also, it was reported that Cor had the inhibitory effect on NSCLC cells (Wei et al., 2019). However, the effects of Cor in reversing DDP resistance in NSCLC are unclear.

AMP-activated protein kinase (AMPK), as a stress-response molecule, is closely associated with tumor-suppressive functions by suppressing the activity of Akt and downstream mammalian target of rapamycin (mTOR), leading to cell growth inhibition and cell cycle arrest (Wang et al., 2016). Meanwhile, AMPK activation could inhibit p53 and p27 to exert tumor suppressor function. AMPK has been reported to directly phosphorylate p53 on serine 15, which is the common phosphorylation site of DNA damage response kinases (Jeon et al., 2012). Notably, multiple recent studies proved that AMPK is a major regulator of drug metabolism closely associated with drug resistance (Shirwany and Zou, 2014; Wang et al., 2016). Shell et al. (2008) presented that metformin administration could significantly increase cancer chemosensitivity associated with mTOR inhibition following AMPK activation. Previous studies indicate that dysregulation of AKT is a prominent feature of various tumors including NSCLC (Lee et al., 2002; Liao et al., 2019). In addition, *AKT* amplification and the mTOR signaling pathway also play an important role in mediating DDP resistance in lung cancer (Feng et al., 2019).

The aim of this study is to identify the effect of Cor in combination with DDP on cell proliferation and apoptosis in both DDP-sensitive and DDP-resistant NSCLC cells both *in vivo* and *in vitro*. Furthermore, we explore the underlying mechanisms of acquiring DDP resistance and suggest potential targets of Cor in reversing DDP resistance in NSCLC cells. The present study will provide a potential novel therapeutic strategy for overcoming DDP resistance in patients with NSCLC.

## Materials and Methods

### Cell Culture and Reagents

Human lung cancer cell line A549 was gifted from the State Key Laboratory of Oncology in South China and cultured in RPMI1640 (Gibco, Carlsbad, CA, United States) containing 10% fetal bovine serum (FBS) (Invitrogen Corp., Carlsbad, CA, United States) and 100 U/ml streptomycin/penicillin (Gibco, Carlsbad, CA, United States) at 37°C and 5% CO_2_. A549DDP cells (DDP-resistant human lung cancer cells) were cultured in RPMI 1640 medium containing 10% FBS, penicillin, and streptomycin. Moreover, DDP was added to the medium of A549DDP cells to a final concentration of 2 μg/ml. Cor and DDP were purchased from Sigma (St. Louis, MO, United States) and dissolved in dimethyl sulfoxide (DMSO). Cor was dissolved in DMSO to make a 1 mM stock solution and stored at −20°C until use. DDP was dissolved in physiological saline to make a 10 mM stock solution and stored at −20°C until use.

### Cell Viability Assay

The cell viability of NSCLC cells treated with Cor and DDP was measured by CCK-8 kit (Dojindo Laboratories, 119 Kumamoto, Japan). A549 and A549DDP (8.0 × 10^3^ cells per well) were seeded into 96-well plates. Cells were treated with different concentrations of Cor and DDP. After 24 h, 10 μl of CCK-8 solution was added into each well and incubated for 2 h and cell viability was determined by a microplate reader at 450 nm (Thermo Scientific, Rockford, IL, United States). The proliferative inhibition rate was calculated using the formula: proliferative inhibition rate = (1 − experimental group/control group) × 100%. The 50% inhibitory concentration (IC_50_) value was calculated by non-linear regression analysis using SPSS 20.0 software.

### Synergy Determination

The combination index (CI) was determined using isobologram analysis based on the Chou-Talalay method. The derived combination index equation for two drugs: CI = (D)1/(Dx)1 + (D)2/(Dx)2. The CI values represent the modes of interaction between two drugs. CI < 1 indicates synergism, CI = 1 indicates an additive effect, and CI > 1 indicates antagonism.

### Colony Formation Assay

Six hundred cells per well were seeded into six-well plates and cultured for 2 weeks. Then, colonies were fixed in 4% paraformaldehyde and stained with crystal violet. The number of colonies was counted under a microscope.

### Cell Cycle Analysis

Cell cycle distribution was determined by Cell Cycle Detection Kit (4A Biotech Co., Ltd., Beijing, China). After treating the cells with Cor, DDP alone, or in combination for 48 h, cells were harvested and fixed in 70% ethanol overnight at 4°C. Then, cells were incubated with 100 μl of RNase in a 37°C water bath for 30 min followed by staining with 400 μl of propidium iodide (PI) for 30 min at 37°C. At least 50,000 cells from each sample were detected for cell cycle distribution by a flow cytometer (Becton Dickinson, San Jose, CA, United States).

### Cell Apoptosis Assay

Cells were treated with different concentrations of Cor, DDP alone, or in combination for 48 h. Cell apoptosis was detected using an Annexin V-FITC apoptosis detection kit (4A Biotech Co., Ltd., Beijing, China). All samples were analyzed by a flow cytometer (Becton Dickinson, San Jose, CA, United States).

### Western Blotting

Cells were treated with Cor or DDP for 48 h and washed with PBS following lysing in sodium dodecyl sulfate (SDS) lysis buffer at 100°C for 20 min. Lysates were centrifuged at 4°C for 15 min and then the supernatant was collected. Equal lysates were denatured in 10% SDS sample buffer and separated by 10% SDS-polyacrylamide gel electrophoresis and transferred to polyvinylidenedifluoride membranes (0.22 μm, Millipore, MA, United States). The polyvinylidenedifluoride membranes were blocked in 5% skim milk for 1 h and incubated with anti-p-P53, anti-P53, anti-cyclinD1, anti-P27, anti-caspase-3, anti-p-caspase-3, anti-Bcl-2, anti-Bax, anti-AMPK, anti-p-AMPK, anti-mTOR, anti-p-mTOR, anti-S6, anti-p-S6, anti-P70S6K, anti-p-P70S6K, anti-AKT, anti-p-AKT, and anti-GAPDH (1:1000) overnight at 4°C, followed by incubating with horseradish peroxidase-conjugated secondary antibodies (Cell Signaling Technology, Danvers, MA, United States) at room temperature for 1 h. Protein bands were visualized using an enhanced chemiluminescence kit (Beyotime, Shanghai, China) and detected with an electrochemiluminescence system (Thermo Fisher Scientific, MA, United States). The densitometry of the protein bands was measured using ImageJ (NIH image software) and normalized to their relevant controls.

### Potential Target Recognition Based on PharmMapper

PharmMapper^1^ is supported by a large, in-house repertoire of a pharmacophore database extracted from all the targets in TargetBank, DrugBank, Binding DB, and PDTD. PharmMapper contains more than 7000 receptor-based pharmacophore models (covering information related to 1627 drug targets, 459 of which are human protein targets). First, the SDF of RA was downloaded from PubChem Compound^2^ and then uploaded to PharmMapper. Following proper parameter setting, target identification was then carried out, and information regarding the top 300 potential protein targets was obtained.

### RNA-Seq Analysis

Total RNA was extracted using Trizol reagent (Invitrogen, CA, United States) following the manufacturer’s procedure. RNA-seq analysis was completed using IIIumina Hiseq 4000 (LianChuan Sciences, Hangzhou, China). Gene ontology (GO) terms for functional categorization were carried out according to molecular function, biological process, and cellular component ontologies with an *E*-value threshold of 10^–5^. The pathway assignments were performed by sequence searches against the Kyoto Encyclopedia of Genes and Genomes (KEGG) database and using the BLASTX algorithm with an *E*-value threshold of 10^–5^. Fragments per kilobase of exon model per million mapped reads values were used to measure the expression abundance of each assembled transcript. Between the two samples, a minimum of a twofold difference in expression was considered as different expression.

### Measurement of GSH

Intracellular levels of GSH were determined by using GSH-Glo Glutathione Assay Kit (Promega, Madison, WI, United States) according to the manufacturer’s instructions.

### Mouse Xenograft Model

A total of 2 × 10^6^ A549 or A549DDP cells were suspended in 100 μl of normal saline and subcutaneously injected into unilateral axillary fossae of BALB/c nude mice (4 weeks old, *n* = 5 per group). Forty mice were purchased from Charles River Laboratories (Beijing, China) and randomly divided into eight groups for the construction of a subcutaneous tumor xenograft model. The tumor size was measured and the volume was calculated according the formula: 0.5 × length × width^2^. When the volume of tumors reached 450–500 mm^3^, the mice received an equal volume of Cor (15 mg/kg), DDP (1.5 mg/kg), Cor (10 mg/kg), and DDP (1 mg/kg) via intraperitoneal injections twice a week. At the end point of study, mice were sacrificed by cervical dislocation and tumors were excised. The animal experiments were administered according to the guidelines of Institution Animal Care and Use Committee, and all protocols were approved by The First Affiliated Hospital of Sun Yat-sen University (Guangzhou, China).

### Statistical Analysis

All experiments were repeated at least three times. All values are presented as means ± standard deviation (SD). Statistical analysis was performed by the Student’s *t*-test. *P* < 0.05 was considered significant. All statistical analysis was performed using SPSS 22.0 software (Chicago, IL, United States).

## Results

### Cor Reverses Cisplatin Resistance in NSCLC Cells

**FIGURE 1:**
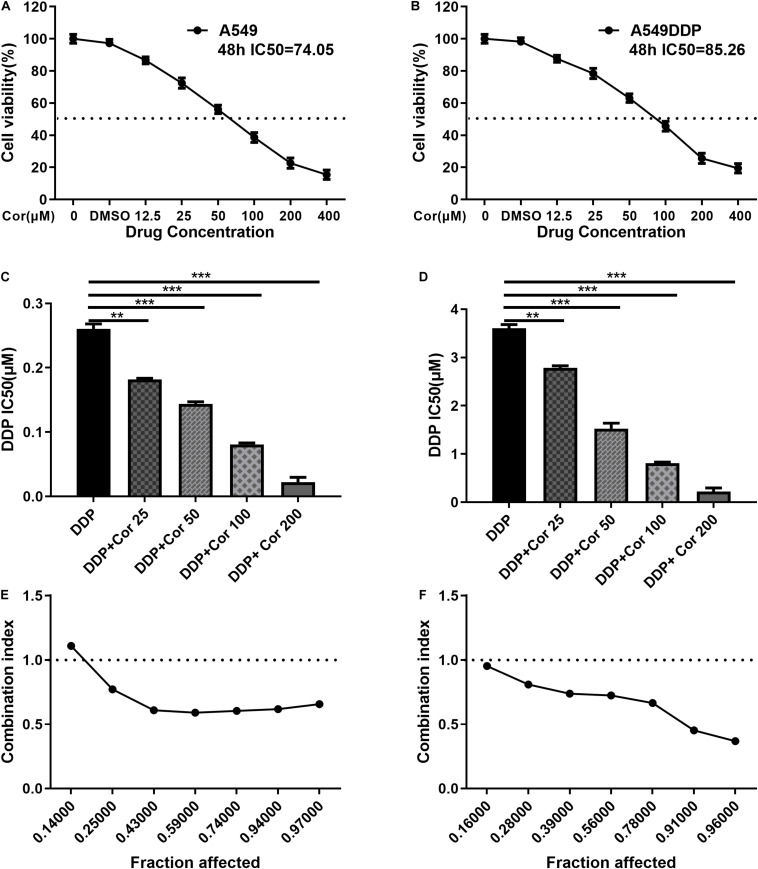
Proliferative inhibitory effect of Cor, DDP and the combination treatment on non-small-cell lung cancer cells (NSCLCs) or DDP-resistant NSCLs. The survival rations of A549 cells **(A)** or A549DDP **(B)** with different Cor concentration were detected by CCK-8 assay. IC50 of DDP was detected for A549 cells **(C)** or A549DDP **(D)** with DDP and different concentration of Cor in combination. Synergistic effects between Cor and DDP were presented as Fa=CI plots for A549 cells **(E)** or A549DDP **(F)**. All data are presented as the mean ± SD of three independent experiments. **p < 0.01; ***p < 0.001 compared to the control.

We have established the A549DDP cell line with persistent DDP resistance and the resistance index (RI) was 11.19 ± 0.50 (Liao et al., 2020). To investigate the effect of Cor on NSCLC cells, we treated A549 and A549DDP cells with various concentrations of Cor for 48 h and measured cell viability by CCK-8 assay. Our data showed that Cor significantly induced concentration-dependent NSCLC cell death, with IC_50_ values of 74.05 μg/ml in A549 cells and 85.26 μg/ml in A549DDP cells (Figures 1A,B). The ability of Cor to inhibit NSCLC cell proliferation was similar between A549 and A549DDP cells. We also found that treatment with combination of Cor and DDP significantly increased the sensitivity of NSCLC cells to DDP (Figures 1C,D). Moreover, the combination index of Cor and DDP is below 1 in both A549 and A549DDP cells, which indicated that the combination of Cor and DDP exerted synergic action in NSCLC cells (Figures 1E,F). These data suggest that Cor has similar effect in inhibiting tumor cell proliferation in both A549 and A549DDP cells, and Cor re-sensitized NSCLC cells to DDP treatment.

### The Combination of Cor and DDP Suppresses Cell Proliferation in NSCLC Cells

**FIGURE 2:**
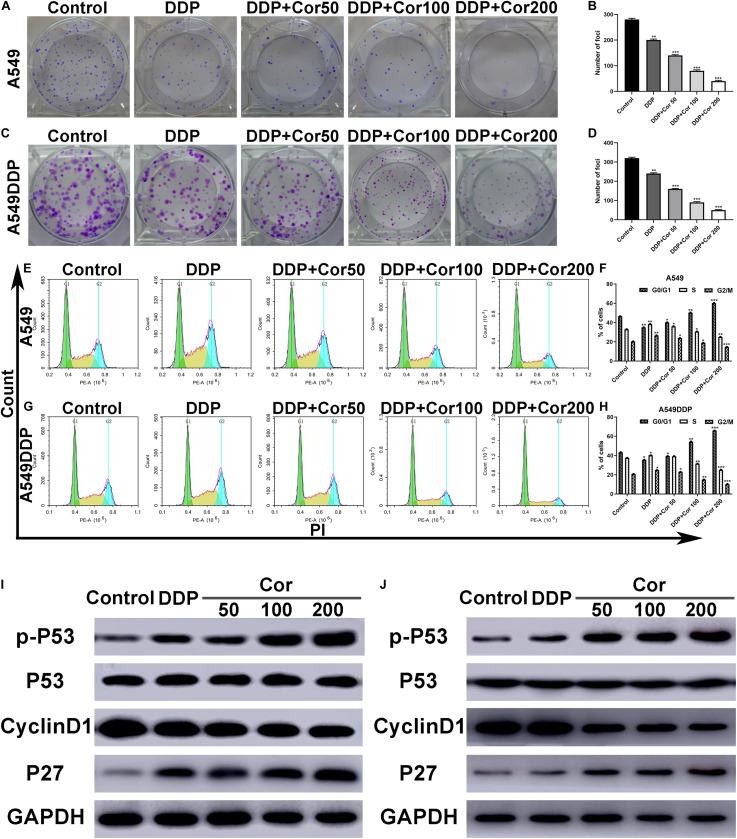
Inhibition of cell proliferation and induction of cell cycle arrest by DDP alone and combined with Cor in NSCLC cell lines. **(A–D)** Cells were treated with 0, 50, 100, and 200 μg/ml Cor combined with 1.5 or 15 μg/ml DDP for 48 h; representative images of A549 **(A,B)** and A549DDP **(C,D)** clone formation are shown. ***p < 0.001 compared with the control. **(E–H)** Cell cycle analysis. Percentages of A549 and A549DDP cells in the G0/G1, S, and G2/M phases are presented, respectively. Effects of DDP and combined with various concentrations of Cor medication on cell cycle distribution. A549 **(E,F)** and A549DDP **(G,H)** cells were treated with 0, 50, 100, and 200 μg/ml Cor combined with DDP for 48 h, and cell cycle distribution was measured by flow cytometry after PI staining. **(I,J)** p-P53, P53, Cyclin D1, and P27 protein levels were determined by Western blot analyses. GAPDH was used as the loading control. All data are presented as the mean ± SD of three independent experiments. *p < 0.05; **p < 0.01; ***p < 0.001 compared to the control.

Colony formation assay and cell cycle study were performed to evaluate the effects of combination of Cor and DDP on proliferation in A549 and A549DDP cells. When Cor and DDP were combined, the efficiency of colony formation of A549 and A549DDP cells was markedly suppressed in a dose-dependent manner as compared to DDP single-treatment groups (Figures 2A–D). Furthermore, we analyzed whether Cor combined with DDP-induced inhibition of cell proliferation was related to cell cycle regulation based on DNA content by flow cytometric analysis. With A549 and A549DDP cells treated with DDP individually, cells in the G0/G1 phase were 35.03% and 35.66%, respectively. With combination treatment with Cor and DDP, the cell cycle arrest of A549 and A549DDP increased in a concentration-dependent manner (Figures 2E–H). Collectively, these results showed that the enhanced DDP treatment effect by Cor was associated with tumor cell cycle arrest.

As P53, cyclinD1, and P27 are G1-S modulators (Xuan et al., 2005), we examined the expression of these cell cycle-regulated proteins by Western blotting. As indicated in Figures 2I,J, the level of p-P53 and P27 increased to some extent, while the level of cyclinD1 decreased in the A549 and A549DDP cells treated with Cor plus DDP in a dose-dependent manner. However, the level of P53 did not alter. Cor promotes the phosphorylation of p53 tumor-suppressor protein to induce NSCLC cell apoptosis. These results are evidence that the synergistic effect of Cor and DDP on cell cycle arrest at G0/G1 is mainly through the key G1-phase regulatory molecules P53, cyclinD1, and P27.

### Cor Enhances Apoptotic Effect of DDP on NSCLC Cells

**FIGURE 3:**
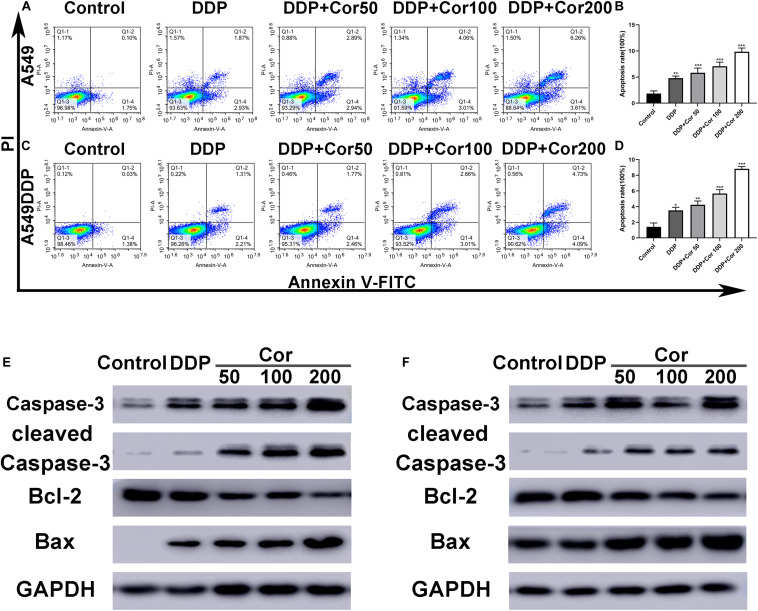
Induction of apoptosis by DDP with or without Cor in NSCLC cell lines. Induction of apoptosis with DDP or combined with different concentration of Cor in both A549 **(A,B)** and A549DDP **(C,D)** cell lines using Annexin-V-FITC/PI staining. Western blot analysis of caspase-3, cleaved caspase-3, Bcl-2 and Bax and GAPDH was used as control in A549 **(E,F)** or A549DDP cell lines treated with DDP or combination between DDP and different concentration of Cor. All data are presented as the mean ± SD of three independent experiments. *p < 0.05; **p < 0.01; ***p < 0.001 compared to the control.

To further assess the effects of Cor in combination with DDP, we incubated cells with DDP alone or with Cor to investigate the effect of the two drugs on cell apoptosis by Annexin V-FITC/PI staining and Western blotting. Cell apoptosis induced in a dose-dependent manner when cells were incubated with the combination of Cor and DDP. In comparison with DDP, the combination of Cor and DDP had a greater effect on cell apoptosis in A549 and A549DDP cells (Figures 3A–D). These results demonstrated that DDP combined with Cor has better apoptotic effect on NSCLC cells than single treatment with these drugs.

The Bcl-2 family proteins including both pro- and anti-apoptotic proteins are key regulators in the mitochondrial apoptotic pathway. The low expression of Bcl-2, a founder member of the Bcl-2 family of apoptosis regulator proteins, leads to apoptosis by activating pro-apoptotic protein Bax oligomerization to releasing cytochrome c into the cytosol and activates caspases including caspase-3 to cause cascade reactions that cleave essential proteins complement throughout the cell (Ola et al., 2011). Western blot assay revealed that the amounts of cleaved caspase-3, caspase-3, and pro-apoptotic protein Bax were markedly increased in both A549 and A549DDP cells treated with the two drugs combined as compared with cells treated with DDP alone, while the expression of anti-apoptotic protein Bcl-2 were decreased (Figures 3E,F). These data indicated that the combination of two drugs had a greater effect than DDP treatment individually. The increased cell apoptosis might be caused by upregulation of Bax and downregulation of Bcl-2 to facilitate activation of the caspase cascade.

### GO and KEGG Analyses Identify Potential Proteins and Signaling Pathways Involved in NSCLC Cell Resistance to DDP

**FIGURE 4:**
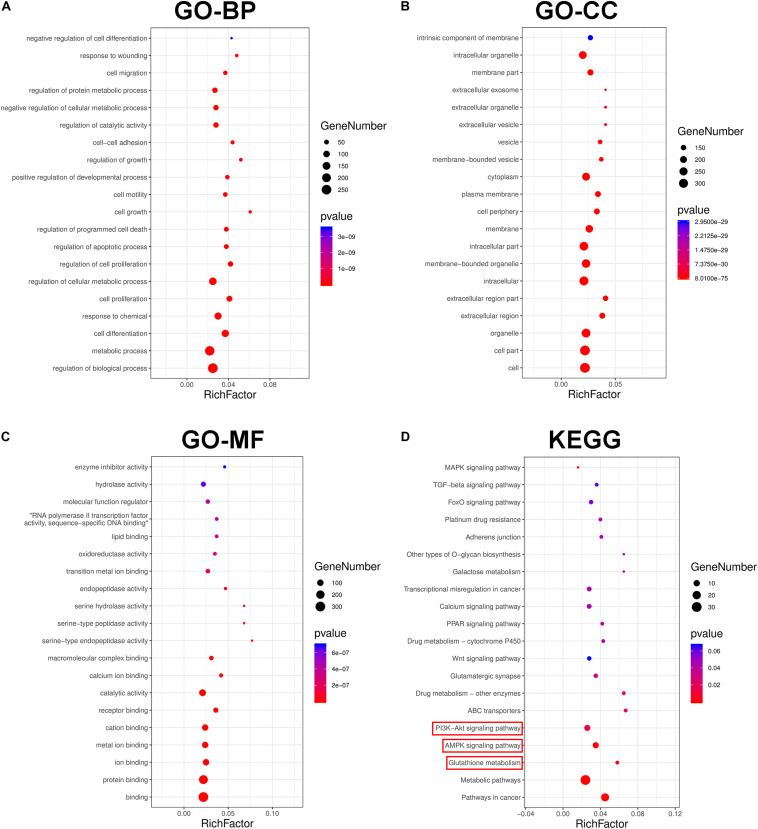
The transcriptional changes between A549 and A549DDP cell lines by GO and KEGG pathway analysis. GO term analysis of differently expression genes (DEGs) in biological process **(A)**, cellular component **(B)** and molecular function **(C)**. **(D)** KEGG pathway analysis of signal transduction pathways involved in NSCLC cell lines resistant to DDP.

RNA sequencing (RNA-Seq) screening revealed differentially expressed genes (DEGs) in DDP-sensitive and DDP-resistant NSCLC cells. GO analysis and pathway enrichment analysis were performed to further understand the functions and signaling pathways involved in NSCLC cells resistant to DDP. Our findings showed that there were 304 differential genes between A549 and A549DDP cells (Supplementary Table 1). GO analyses indicated that these DEGs were associated with biological process (BP), cellular component (CC), and molecular function (MF) (Figures 4A–C). KEGG pathway analysis showed that AMPK and AKT signaling pathways and glutathione metabolism were involved in NSCLC cells resistant to DDP (Figure 4D). These results suggested that the major cause of DDP resistance might be due to the intracellular GSH level as well as AMPK and AKT signaling pathways.

### Cor Induces a Dose-Dependent Reduction in GSH-Mediated DDP Resistance in NSCLC Cells

**FIGURE 5:**
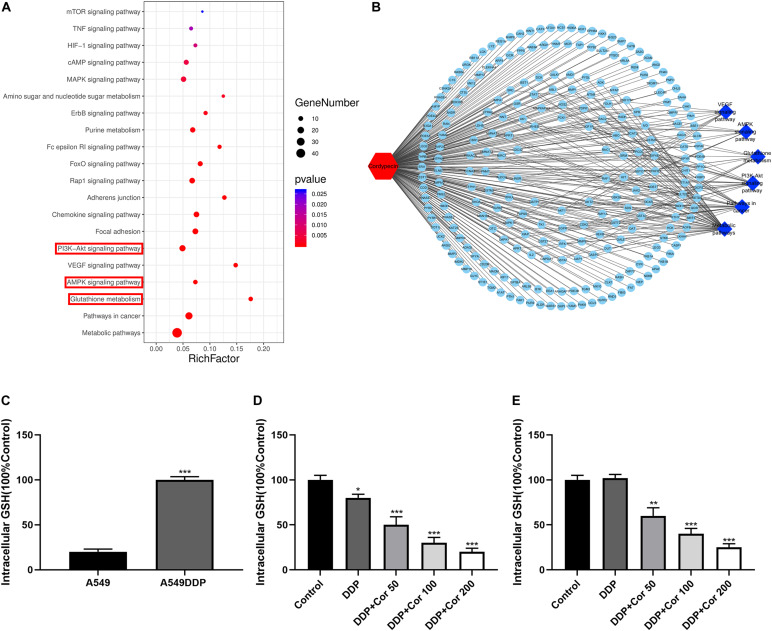
Suppression of intracellular glutathione (GSH) by Cor and DDP in NSCLC cells. **(A)** KEGG pathway analysis of signal transduction pathways involved in Cor treating NSCLC cells. **(B)** Potential targets by Cor in NSCLC cells using Pharma Mapper. **(C)** Measuring the intracellular glutathione in A549 and A549DDP. Intracellular glutathione assay of A549 cells **(D)** or A549DDP **(E)** with 0, 50, 100, 200 μg/mL Cor combined with 1.5 μg/mL or 15 μg/mL DDP for 48 h. All data are presented as the mean ± SD of three independent experiments. *p < 0.05; **p < 0.01; ***p < 0.001 compared to the control.

We obtained 241 top potential targets of Cor using Pharma Mapper, which indicated that Cor had superb druggability with these potential targets (Supplementary Table 2). Combined with KEGG pathway information analysis, we identified glutathione metabolism, AMPK, and AKT signaling pathways to be involved in Cor treatment of NSCLC (Figures 5A,B). We quantified the intracellular glutathione (GSH) level to explore the DDP resistance mechanism of A549DDP cells. GSH expression was significantly higher in the resistant A549DDP cells than in A549 cells (Figure 5C). The increasing intracellular GSH levels may explain the attenuation of DDP-mediated apoptosis effects. This is supported by the demonstration that prominent elevations in GSH levels have been correlated directly with DDP resistance in a panel of tumor models (Kelland, 1993). Our results showed that GSH expression was downregulated in A549 cells but not in A549DDP cells by treatment with DDP alone. However, increased concentration of Cor in combination with DDP and the GSH level were significantly reduced (Figures 5D,E), leading to mitochondrial-mediated apoptosis. These results indicate that upregulation of GSH is an important way for NSCLC cells to become drug resistant. More importantly, Cor might reverse resistance to DDP in NSCLC cells by downregulating the expression of GSH.

### Cor Reverses DDP Resistance via Activation of AMPK and Inactivation of AKT Signaling Pathways in NSCLC Cells

**FIGURE 6:**
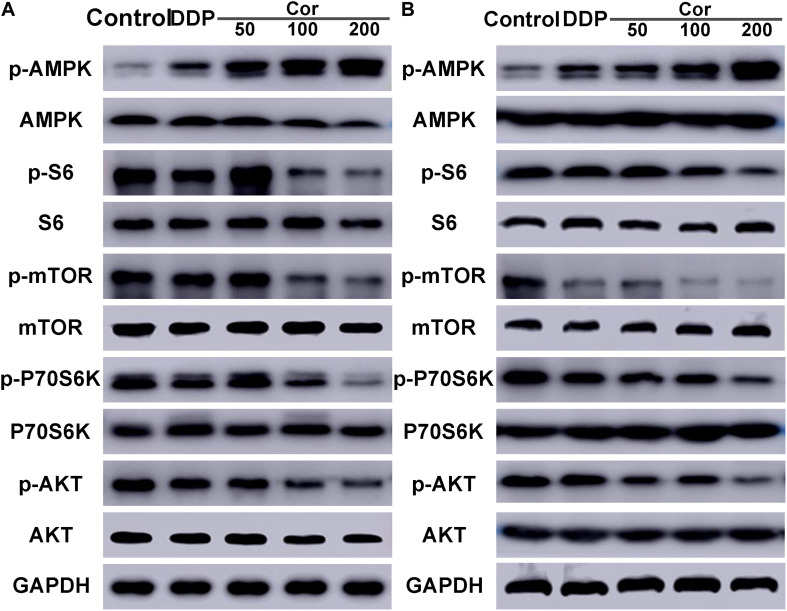
Western blot assay of AMPK, p-AMPK, S6, p-S6, mTOR, p-mTOR, P70S6K, p-P70S6K, AKT, p-AKT in A549 **(A)** and A549DDP **(B)** cell lines treated with DDP or combination between DDP and different concentration of Cor. All data are presented as the mean ± SD of three independent experiments.

Based on the above results, Cor exhibits a significant reverse effect on NSCLC cell resistance to DDP by activating AMPK and inactivating AKT signaling pathways. We further select several target proteins regulating the signaling transduction pathways by Western blotting in order to verify the reliability of the bioinformatics data. After 48 h of drug treatment, DDP promoted phosphorylation of AMPK but did not significantly affect total amount of proteins including AMPK, AKT, mTOR, P70S6K, and S6. Cor, in combination with DDP, not only upregulated AMPK phosphorylation but also reduced p-AKT, p-mTOR, and downstream p-P70S6K and p-S6 in both A549 and A549DDP cells compared with DDP single treatment in a dose-dependent manner (Figures 6A,B). Moreover, the increased sensitivity was seen in both DDP-sensitive and -resistant cell lines, drawing the rational conclusion that the combinational treatment of Cor and DDP affected the AMPK and AKT signaling pathways. Our results suggested that Cor treatment activated AMPK and inhibited the activity of AKT, which associates with phosphorylates mTOR, thereby depressing the phosphorylation of S6K to suppress cell proliferation.

### Cor Combined With DDP Potentiates Tumor Regression in a NSCLC Xenograft Mouse Model

**FIGURE 7:**
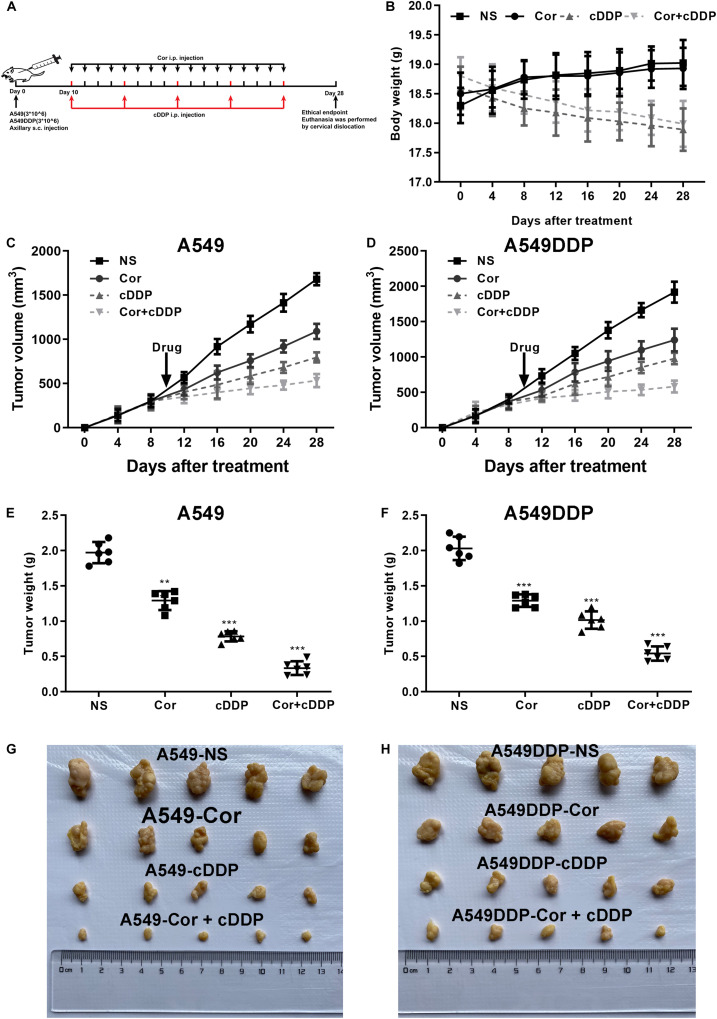
The effect of DDP or Cor alone or combined between DDP and Cor compared with control on A549 or A549DDP cells xenograft tumor growth. **(A)** The timeline of A549 or A549DDP cells incubation subsequently treated with drugs. **(B)** Time course of mice body weight. Time course of A549 **(C)** or A549DDP **(D)** cells tumor growth. Dots graphs represent the weight of the A549 **(E)** or A549DDP **(F)** at the indicated time point of different treatments. Visual comparison of the A549 **(G)** or A549DDP **(H)** dissected tumor tissues. All data are presented as the mean ± SD of three independent experiments. **p < 0.01; ***p < 0.001 compared to the control.

To further determine the efficacy of Cor and DDP combination treatment on tumor regression *in vivo*, A549 or A549DDP cells were implanted subcutaneously into nude mice. After tumor attained a size of 450 to 500 mm^3^, mice were treated with drugs twice a week as mentioned in the “Materials and Methods” Section (Figure 7A). Xenograft tumors grew slower in combination of Cor and DDP-treated group compared to that in the control group (Figures 7C,D). While administration of Cor or DDP alone marginally inhibited tumor growth in comparison with the control group, a combination of Cor + DDP administration greatly regressed the tumor volume (Figures 7E–H). We also noted that the health of the mice was not compromised by Cor treatment (Figure 7B). Our data suggested that Cor combined with DDP can significantly inhibit tumor growth compared with Cor or DDP single treatment. These results indicated that Cor not only reverses NSCLC resistance to DDP but also alleviates DDP-induced weight loss.

## Discussion

Cisplatin is the first member of a class of platinum-containing anticancer drugs, which causes DNA cross-linking to trigger cell apoptosis (Apps et al., 2015). Although DDP is a commonly used anticancer agent, drug resistance greatly limits its clinical usefulness. Thus, it is important to explore combined strategies to improve the therapeutic effect of DDP on NSCLC. In the present study, we demonstrated that Cor and DDP were synergistic at inhibiting cell proliferation and inducing apoptosis of NSCLC in the presence or absence of DDP resistance. Moreover, the AMPK and AKT signaling pathways as well as glutathione metabolism were involved in attenuation of DDP resistance in NSCLC cells by Cor and DDP combination treatment.

The mechanisms contributing to DDP resistance include DDP damage/repair proteins, prevention of drug to reach DNA target, and activating signaling pathways to prevent apoptosis (Siddik, 2003). Overexpression of apoptotic inhibitor molecules directly or indirectly attenuates the activation of caspase cascade contributing to DDP resistance (Henkels and Turchi, 1999). Hayakawa et al. (2000) reported that HER-2 overexpression involved in DDP resistance is due to inactivation of the pro-apoptotic protein Bad following its phosphorylation by Akt. Several measures have been proposed to decrease DDP resistance. Inhibition of miR-196a is revealed to be able to restore sensitivity to DDP in lung cancer via downregulation of drug-resistant protein expression and inhibition of drug efflux (Li et al., 2016). mTOR inhibitor (CCI-779) appears to reverse DDP resistance by increasing the growth inhibition and enhancing the apoptotic effect in DDP-resistant cells (Wu et al., 2005). Rosmarinic acid reverses NSCLC resistant to DDP by activating the MAPK signaling pathway (Liao et al., 2020). Our results of GO and KEGG analysis indicated that AMPK and AKT signaling pathways are involved in NSCLC cells resistant to DDP, which provided potential targets for overcoming DDP resistance.

Cor, as a traditional Chinese medicine, plays an important role in the treatment of cancers including inhibiting proliferation of tumors and reducing toxicity of conventional therapy (Lichti-Kaiser and Staudinger, 2008). It is noteworthy that Cor can reverse drug resistance, especially used in combination with other chemotherapeutic drugs. Several combination therapies using Cor with other chemotherapeutic drugs have been explored in different cancers. Cor combined with temozolomide synergistically inhibits glioma cells via AMPK signaling pathway (Bi et al., 2018). The combination treatment of Cor and DDP shows an enhanced apoptotic effect on OC3 human oral cancer cells through the JNK/caspase-7 signaling pathway (Chen et al., 2014). Wei et al. (2019) confirmed that Cor in combination with gefitinib can be used as a new therapeutic strategy for gefitinib-resistant lung cancer. It is reasonable to suggest that there are different mechanisms underlying the pharmacological effect of combination with Cor and other chemotherapeutic drugs in different types of cancers. Therefore, we investigated the effect of Cor in combination with DDP on reducing DDP resistance in NSCLC and explored the possible underlying mechanisms. We demonstrated that the combination of Cor and DDP had synergistic effect on proliferation inhibition and apoptosis induction in A549 and A549DDP cells. These findings indicated that Cor might enhance DDP treatment in both sensitive and resistant NSCLC cells. Hence, a combination of Cor and DDP provides a novel approach in the treatment of NSCLC especially for DDP-resistant patients.

Based on DEGs between A549 and A549DDP cells in RNA-Seq, we found that glutathione metabolism, AMPK, and AKT signaling pathways might play major roles in NSCLC cells resistant to DDP. Furthermore, we found that the effect of Cor in the treatment of NSCLC was mediated though reducing glutathione metabolism, activation of AMPK, and inhibition of AKT signaling pathways based on Pharma Mapper analysis of 241 potential protein targets for Cor. On the one hand, our results showed that intracellular GSH in A549DDP was significantly increased compared to that in A549 cells, which is inconsistent with reports in a number of cisplatin-resistant tumor models (Kelland, 1993). It is generally accepted that the increased conjugation reaction between GSH and cisplatin is a significant factor of drug resistance (Siddik, 2003). Studies have demonstrated that GSH elevated by cisplatin contributes to increase DNA repair or increase the inhibitory effect on apoptosis (Kelland, 1993; Slater et al., 1995). Our results also showed that GSH as well as Bcl-2 was overexpressed in A549DDP cells. It is inconsistent with reports that higher intracellular GSH levels have correspondingly overproduced the Bcl-2 protein, which may associate with the anti-apoptotic functions of Bcl-2 (Hockenbery et al., 1993; Chiao et al., 1995). Conversely, downregulation of GSH levels by Cor potentiated DDP cytotoxicity, which might in fact increase the Bax:Bcl-2 ratio as well as activation of caspase cascade contributing to cell apoptosis. Members of the Bcl-2 family localized in the mitochondria are key players involved in regulating apoptosis (Hanahan and Weinberg, 2000). This understanding is consistent with overexpression of *bcl-2* that is facilitated by DDP resistance, and this is likely associated with an increase in GSH levels (Chiao et al., 1995). On the other hand, our results showed that the attenuation of DDP resistance was associated with upregulation of p-P53. Studies have proposed that several genes transactivated by p53 as a result of Cor and DDP combination exposure are associated with DNA damage, cell cycle arrest, and apoptosis (Delmastro et al., 1997; Hershberger et al., 2002). When DNA damage overwhelms cellular repair capacity, the well-orchestrated cell apoptosis activated by Cor and DDP induced Bax translocated from the cytosol to the mitochondria, where cytochrome *C* activates the caspase 3 pathway and leads to apoptosis (Makin et al., 2001). Our results indicate that Cor-mediated apoptosis of NSCLC cells is associated with the decreased expression of Bcl-2, accompanied by overexpression of Bax and p53.

It should be noted that many of the studies that define the apoptotic function of p53 is dependent on several DDP-induced upstream signaling pathways that activate the tumor-suppressor protein by upregulating this phosphorylation (Fritsche et al., 1993; Feng et al., 2019). AMPK activation is also revealed to cause cell cycle arrest associated with stabilization of p53 and the cyclin-dependent kinase inhibitor p27 (Wang et al., 2016). It is reported that a lack of p53 phosphorylation due to defective upstream activation of AMPK is the probable mechanism contributing to drug resistance (Wang et al., 2016). In this regard, the AMPK protein is activated by Cor and DDP, contributing to activate the tumor-suppressor activator p53. Once the AMPK is activated by the combination of Cor and DDP, downstream signaling either is propagated through the p53 pathway, which in turn activates the apoptosis-related proteins such as Bax and the cell cycle-related proteins including p27, or suppresses the mTOR/S6K pathway. Basal activity of the AMPK pathway facilitates the induction of Bax and P27 by Cor and DDP in a p53-dependent manner. Meanwhile, AMPK amplification is revealed to reduce expression of the downstream mTOR, which is an essential component of the mTOR/p70S6K1 pathway (Hawley et al., 2005). Cor was found to inhibit NSCLC cell proliferation by decreasing phosphorylation of 70S6K1 and S6, which was greatly associated with mTOR inhibition following AMPK activation. As a result, Cor reversed resistance of NSCNC cells to DDP through upregulating expression of AMPK related to the mTOR/p70S6K1 pathway to reduce cell proliferation.

We then unveiled that Cor increased sensitivity in both DDP-sensitive and -resistant cells related to inactivate the AKT pathway. In many tumors, the protein kinase Akt is hyperactivated and p-Akt is the activated form of Akt that has a biological function. Akt has been reported to be a negative regulator of AMPK and an upstream positive regulator of mTOR (Wang et al., 2016). Once activated, Akt activation of the mTOR pathway leads to increased cell proliferation and reduced cell apoptosis involved in the pathogenesis of cancers (Siddik, 2003). The AKT and mTOR constitutive activation confers drug resistance to many types of cancers, including NSCLC. Activated downstream protein S6 in the mTOR signaling pathway often regulates cell proliferation and survival, thus diminishing the inhibitory effect of DDP on NSCLC, with resultant resistance to DDP. As results above show, AMPK and AKT signaling pathways are intimately associated with DDP resistance. In contrast, Cor appears following perturbation of the pathways by dysfunction of Akt and activation of AMPK, which are upstream regulators of mTOR. This perturbation in the two pathways is consistent with the finding that Cor effectively inhibits cellular growth due to mTOR inhibition via AMPK and AKT signaling pathway (Bi et al., 2018).

**FIGURE 8:**
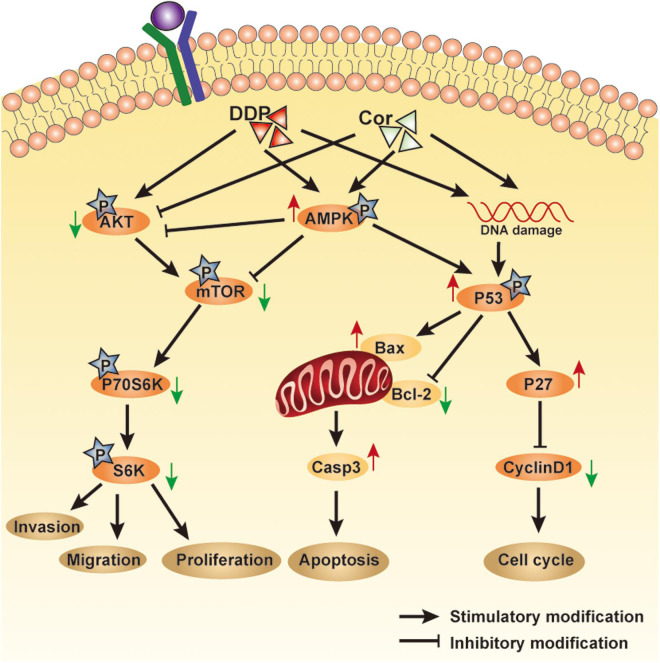
An overview of pathways involved in mediating Cor and DDP-induced cellular effects. Cell proliferation or cell apoptosis will depend on the relative intensity of the signals generated and the crosstalk between the pathways involved.

Taken together, we demonstrated that Cor and DDP had a synergistic effect on inhibiting proliferation and inducing apoptosis in the DDP-sensitive and -resistant NSCLC cells. Cor enhanced DDP therapeutic efficacy but more importantly reversed DDP resistance of NSCLC associated with upregulation of AMPK and inactivation of AKT signaling pathways (Figure 8). Our findings suggested that it is worthwhile to explore the potential application of Cor in combination with DDP in the treatment for NSCLC, especially for DDP-resistant patients.

## Data Availability Statement

The raw data supporting the conclusions of this article will be made available by the authors, without undue reservation.

## Ethics Statement

The animal study was reviewed and approved by Institutional Animal Care and Use Ethics Committee of First Affiliated Hospital of Sun Yat-sen University.

## Author Contributions

L-ZL and M-FH designed the research study. X-ZL, YG, and MZ performed the majority of the experiments and data analysis. L-LS and L-TT contributed to specimen preparation. J-XZ, WG, H-RC, C-YG, and Z-WX assisted with *in vivo* experiments. X-ZL and YG wrote the manuscript. All authors read and approved the final version of the manuscript.

## Conflict of Interest

The authors declare that the research was conducted in the absence of any commercial or financial relationships that could be construed as a potential conflict of interest.

## Footnotes

1 http://lilab.ecust.edu.cn/pharmmapper/index.php

2 https://www.ncbi.nlm.nih.gov/pccompound/
