# Supplementary material for: Cordycepin Reverses Cisplatin Resistance in Non-small Cell Lung Cancer by Activating AMPK and Inhibiting AKT Signaling Pathway
Source: Front Cell Dev Biol. 2021 Jan 15;8:609285. doi: 10.3389/fcell.2020.609285 (PMC7843937; doi:10.3389/fcell.2020.609285)
Supplement: Supplementary file 1 [file Table_1.DOCX]

**Supplement table1 Differential genes between A549 and A549DDP in RNASeq**

| **GENE** | **A549** | **A549DDP** | **GENE** | **A549** | **A549DDP** |
| --- | --- | --- | --- | --- | --- |
| IGFBP7 | 1.008188 | 15841.28 | ELF3 | 7508.988 | 184.4893 |
| MRC2 | 1.008188 | 9947.545 | PCDH7 | 6815.354 | 162.668 |
| VIM | 16.13102 | 84799.62 | C10orf10 | 3080.016 | 61.49644 |
| BICC1 | 2.016377 | 10472.25 | PLTP | 3970.246 | 78.35837 |
| PDE10A | 1.008188 | 4960.382 | CD24P4 | 7213.588 | 133.9035 |
| CNTNAP3B | 1.008188 | 4898.886 | LYPD3 | 3525.635 | 63.48019 |
| TMEM178B | 1.008188 | 4498.167 | SHROOM3 | 6123.737 | 107.1228 |
| CRMP1 | 1.008188 | 4211.514 | TMC4 | 4285.809 | 65.46395 |
| GFRA1 | 1.008188 | 4181.758 | BHLHE41 | 5875.722 | 85.30151 |
| FAM171A1 | 1.008188 | 3738.388 | SYTL2 | 2116.188 | 29.75634 |
| DPYSL3 | 1.008188 | 3541.005 | CAPG | 2925.763 | 40.667 |
| CYP24A1 | 3.024565 | 9595.428 | C1orf106 | 2738.24 | 35.70761 |
| ITGB3 | 2.016377 | 6289.499 | SCNN1A | 6714.535 | 82.32588 |
| SLIT3 | 1.008188 | 3038.122 | TSPAN13 | 6538.102 | 75.38273 |
| FBN2 | 2.016377 | 5784.633 | INA | 2902.575 | 31.7401 |
| NRG1 | 1.008188 | 2758.413 | FAM46C | 2187.769 | 23.80507 |
| COL27A1 | 1.008188 | 2118.651 | SOX15 | 1765.338 | 18.84568 |
| EMB | 2.016377 | 4227.384 | JPH2 | 2240.195 | 21.82132 |
| C12orf75 | 1.008188 | 2046.244 | MAPK13 | 5850.518 | 55.54517 |
| UBE2M | 1.008188 | 2043.269 | WNT6 | 2318.833 | 21.82132 |
| PALM | 1.008188 | 1892.503 | SPINT2 | 16479.85 | 151.7573 |
| HAVCR1 | 2.016377 | 3637.217 | LRG1 | 1480.021 | 12.89441 |
| GALNT14 | 1.008188 | 1622.712 | GALNT3 | 2745.297 | 23.80507 |
| SYNE1 | 4.032754 | 6438.28 | ZNF22 | 1667.544 | 13.88629 |
| KCNMA1 | 4.032754 | 6013.757 | MPZL2 | 1584.872 | 12.89441 |
| AXL | 17.1392 | 24514.27 | F3 | 5896.894 | 47.61015 |
| MMP2 | 1.008188 | 1418.386 | CRABP2 | 3962.181 | 29.75634 |
| DSC3 | 1.008188 | 1412.434 | EGLN3 | 1983.107 | 14.87817 |
| PNMA2 | 5.040942 | 6833.048 | ARHGDIB | 1224.949 | 8.926902 |
| RBMS3 | 1.008188 | 1362.84 | PLEKHA6 | 8964.812 | 63.48019 |
| ANPEP | 1.008188 | 1236.872 | FAM49A | 1686.699 | 11.90254 |
| USP9Y | 2.016377 | 2449.939 | ABCA1 | 2515.43 | 15.87005 |
| TSPAN18 | 1.008188 | 1101.977 | CALB2 | 2348.071 | 13.88629 |
| FHL1 | 7.057319 | 7710.86 | ABCG2 | 2242.211 | 11.90254 |
| WDR72 | 1.008188 | 1028.578 | GSTM3 | 2651.536 | 13.88629 |
| KDM5D | 1.008188 | 1005.764 | GLUL | 4811.075 | 24.79695 |
| RIMS4 | 1.008188 | 1005.764 | CFB | 1545.553 | 7.935024 |
| SPOCK1 | 1.008188 | 1005.764 | GPX2 | 2153.491 | 10.91066 |
| ARMCX3 | 3.024565 | 3015.309 | S100P | 3191.925 | 15.87005 |
| AASS | 1.008188 | 980.9674 | TMPRSS4 | 2232.129 | 10.91066 |
| CPS1 | 2.016377 | 1954 | CLDN4 | 12034.75 | 58.5208 |
| CD38 | 1.008188 | 976.008 | BICDL2 | 1635.282 | 7.935024 |
| CYFIP2 | 5.040942 | 4853.259 | TIMP3 | 2876.362 | 13.88629 |
| NID1 | 4.032754 | 3882.211 | SEMA5A | 1455.824 | 6.943146 |
| FGF2 | 5.040942 | 4768.95 | VGLL3 | 1361.054 | 5.951268 |
| EFNB3 | 1.008188 | 953.1948 | RAC2 | 1362.063 | 5.951268 |
| WIPF1 | 2.016377 | 1871.674 | ANK3 | 2316.817 | 9.91878 |
| USP32P1 | 1.008188 | 933.3572 | INHBB | 9175.523 | 38.68324 |
| APBB1 | 2.016377 | 1830.015 | BST2 | 953.7463 | 3.967512 |
| CENPBD1P1 | 1.008188 | 909.5522 | TINAGL1 | 8401.235 | 34.71573 |
| RFLNB | 2.016377 | 1790.34 | ADORA1 | 1225.957 | 4.95939 |
| SDK1 | 1.008188 | 880.7877 | GLB1L2 | 1266.285 | 4.95939 |
| HNF1B | 2.016377 | 1678.258 | VAV3 | 1270.317 | 4.95939 |
| LHFP | 1.008188 | 828.2182 | CP | 1023.311 | 3.967512 |
| TMEM98 | 1.008188 | 821.275 | SERPINB5 | 1211.843 | 3.967512 |
| RPS4Y1 | 8.065508 | 6544.411 | CDH3 | 3667.79 | 11.90254 |
| TXLNGY | 1.008188 | 816.3156 | AIM1 | 12860.45 | 41.65888 |
| REEP2 | 2.016377 | 1625.688 | NFE4 | 928.5416 | 2.975634 |
| CADM1 | 1.008188 | 812.3481 | AKR1C1 | 23263.95 | 74.39085 |
| TMEM59L | 1.008188 | 793.5024 | FAM83B | 2061.745 | 5.951268 |
| IL32 | 2.016377 | 1568.159 | FXYD3 | 4976.418 | 13.88629 |
| FAM196B | 1.008188 | 779.6161 | DENND1C | 768.2396 | 1.983756 |
| GJA1 | 13.10645 | 10081.45 | FAAH | 844.8619 | 1.983756 |
| RIMS2 | 1.008188 | 739.941 | DIO2 | 4249.514 | 9.91878 |
| UNC13A | 5.040942 | 3626.306 | GALNT6 | 4713.281 | 10.91066 |
| ALPK2 | 2.016377 | 1448.142 | UGT1A6 | 3437.923 | 7.935024 |
| CYS1 | 1.008188 | 700.2659 | CBLC | 887.2059 | 1.983756 |
| CYBA | 7.057319 | 4871.113 | ALDH3B2 | 4262.621 | 8.926902 |
| FOXA2 | 1.008188 | 687.3715 | MUC4 | 6638.921 | 13.88629 |
| INTS6L | 1.008188 | 687.3715 | SLC52A3 | 952.7381 | 1.983756 |
| NSG1 | 1.008188 | 685.3877 | IRF6 | 2214.99 | 3.967512 |
| ZEB1 | 3.024565 | 2001.61 | ESRP2 | 2890.476 | 4.95939 |
| CDH4 | 19.15558 | 12571.06 | H19 | 4702.191 | 7.935024 |
| PRAME | 7.057319 | 4561.647 | MEST | 2425.701 | 3.967512 |
| PTPRN2 | 2.016377 | 1271.588 | FUT9 | 1217.892 | 1.983756 |
| ENSG224078 | 4.032754 | 2493.581 | CARD11 | 3690.978 | 5.951268 |
| SCN8A | 2.016377 | 1217.034 | AGR2 | 3995.451 | 5.951268 |
| SH3BGRL | 7.057319 | 4237.303 | SH3TC2 | 2012.344 | 2.975634 |
| SMO | 3.024565 | 1710.99 | TMEM139 | 1362.063 | 1.983756 |
| TTC28 | 6.049131 | 3188.888 | GRAMD2 | 3458.086 | 4.95939 |
| ZNF618 | 4.032754 | 2014.504 | AKR1C2 | 22028.92 | 29.75634 |
| QPCT | 2.016377 | 1001.797 | TRIM2 | 2968.107 | 3.967512 |
| CACNA1G | 3.024565 | 1381.686 | MDFI | 744.0431 | 0.991878 |
| MSRB3 | 5.040942 | 2279.336 | TNS4 | 772.2724 | 0.991878 |
| AGAP2 | 3.024565 | 1277.539 | ENSG273108 | 777.3133 | 0.991878 |
| LIMD2 | 4.032754 | 1656.436 | LAMA4 | 778.3215 | 0.991878 |
| GRK3 | 2.016377 | 761.7623 | LAD1 | 3140.507 | 3.967512 |
| NFASC | 6.049131 | 2256.523 | OVOL1 | 795.4607 | 0.991878 |
| GCNT3 | 7.057319 | 2462.833 | RAB17 | 800.5016 | 0.991878 |
| KIF5C | 8.065508 | 2770.315 | VAMP8 | 2546.684 | 2.975634 |
| HOXB9 | 3.024565 | 1003.781 | TXNIP | 9347.923 | 10.91066 |
| GXYLT2 | 3.024565 | 998.8212 | PRDX2 | 5145.794 | 5.951268 |
| EVC | 5.040942 | 1611.802 | BSPRY | 866.0339 | 0.991878 |
| TGFB1I1 | 13.10645 | 4159.936 | SHISA9 | 866.0339 | 0.991878 |
| EVA1C | 5.040942 | 1536.419 | PRSS21 | 870.0666 | 0.991878 |
| F2R | 11.09007 | 3225.587 | CLDN3 | 874.0994 | 0.991878 |
| C16orf45 | 9.073696 | 2175.189 | MUC20 | 2649.519 | 2.975634 |
| ADGRB1 | 11.09007 | 2633.436 | NNT | 903.3369 | 0.991878 |
| RARB | 14.11464 | 3281.133 | EPCAM | 10074.83 | 10.91066 |
| ANTXR1 | 98.80247 | 21256.94 | SLC1A3 | 1869.181 | 1.983756 |
| RFTN1 | 13.10645 | 2811.974 | CST6 | 944.6726 | 0.991878 |
| IFI27L2 | 6.049131 | 1287.458 | C1orf116 | 981.9756 | 0.991878 |
| H2AFY2 | 10.08188 | 2100.798 | ARHGEF5 | 1008.188 | 0.991878 |
| LRRC4B | 12.09826 | 2471.76 | FAM83A | 2039.565 | 1.983756 |
| FRAS1 | 7.057319 | 1404.499 | AKR1B10 | 51891.46 | 47.61015 |
| LIX1L | 13.10645 | 2396.377 | TMEM30B | 1101.95 | 0.991878 |
| SOGA3 | 6.049131 | 1087.098 | FUT3 | 1134.212 | 0.991878 |
| NECTIN3 | 7.057319 | 1173.392 | S100A14 | 7070.426 | 5.951268 |
| C14orf159 | 9.073696 | 1471.947 | SLC1A6 | 1179.581 | 0.991878 |
| PCDH9 | 20.16377 | 3267.246 | GATA2-AS1 | 1192.687 | 0.991878 |
| ATP8B2 | 26.2129 | 4085.546 | KRT19 | 103593.4 | 84.30963 |
| PCLO | 9.073696 | 1389.621 | SPINT1 | 4970.369 | 3.967512 |
| CMTM3 | 51.41761 | 7620.599 | CPAMD8 | 1249.146 | 0.991878 |
| RASSF2 | 12.09826 | 1775.462 | PROM2 | 8835.764 | 6.943146 |
| CACNA1H | 20.16377 | 2649.306 | APOD | 1292.498 | 0.991878 |
| TPM2 | 56.45855 | 7257.572 | ANXA8L1 | 1301.571 | 0.991878 |
| FAM129A | 16.13102 | 2052.196 | MIR205HG | 4008.557 | 2.975634 |
| PDGFC | 17.1392 | 2144.44 | ALOX5 | 4080.139 | 2.975634 |
| TCF7 | 13.10645 | 1620.729 | ST14 | 5759.781 | 3.967512 |
| JPH3 | 19.15558 | 2337.857 | ANXA8 | 1443.726 | 0.991878 |
| APLP1 | 33.27022 | 3817.739 | C6orf132 | 1599.995 | 0.991878 |
| TLE4 | 16.13102 | 1677.266 | ESRP1 | 6594.561 | 3.967512 |
| CNTNAP3 | 41.33573 | 4198.62 | TACSTD2 | 12290.83 | 6.943146 |
| DUSP1 | 105.8598 | 10555.57 | INHBA | 5470.431 | 2.975634 |
| KIRREL3 | 26.2129 | 2342.816 | B3GNT3 | 3793.813 | 1.983756 |
| PROS1 | 39.31935 | 3489.427 | PRR15 | 1912.534 | 0.991878 |
| PAPPA | 35.2866 | 3031.179 | BPIFB1 | 1983.107 | 0.991878 |
| TRNP1 | 55.45037 | 4662.819 | EPPK1 | 4165.835 | 1.983756 |
| SGCB | 27.22109 | 2277.352 | LINC00942 | 2135.343 | 0.991878 |
| KLF2 | 29.23747 | 2432.085 | FBP1 | 2210.957 | 0.991878 |
| IGFBP4 | 67.54863 | 5562.452 | RAB25 | 2248.26 | 0.991878 |
| DOCK4 | 44.36029 | 3412.06 | EHF | 6821.403 | 2.975634 |
| ANGPTL4 | 127.0317 | 9520.045 | SYK | 2442.841 | 0.991878 |
| IFITM2 | 45.36848 | 3378.337 | CYP4F11 | 4929.033 | 1.983756 |
| TUBA1A | 140.1382 | 9904.894 | MAGEA6 | 2525.512 | 0.991878 |
| EMP3 | 56.45855 | 3990.325 | S100A9 | 20755.58 | 7.935024 |
| FBN1 | 57.46674 | 3570.761 | KLK5 | 7904.198 | 2.975634 |
| AHNAK2 | 625.0769 | 36817.52 | CDH1 | 13325.23 | 4.95939 |
| GLIS3 | 74.60595 | 3908.991 | UCA1 | 2726.142 | 0.991878 |
| DLC1 | 128.0399 | 5959.203 | VGLL1 | 5572.258 | 1.983756 |
| COL4A2 | 1161.433 | 49233.85 | OLFML3 | 17287.41 | 5.951268 |
| COL4A1 | 553.4955 | 23261.52 | MACC1 | 2967.099 | 0.991878 |
| LOXL2 | 799.4935 | 32342.17 | CLDN7 | 9119.065 | 2.975634 |
| AKAP12 | 1252.17 | 40293.06 | SLPI | 3375.415 | 0.991878 |
| DCBLD2 | 2208.941 | 48220.15 | GRHL2 | 3768.608 | 0.991878 |
| ABCC2 | 13869.65 | 585.208 | STEAP4 | 4030.737 | 0.991878 |
| AKR1C3 | 24352.79 | 960.1379 | NEURL1 | 4128.532 | 0.991878 |
| SCD | 138438.4 | 5090.318 | ALPP | 4154.745 | 0.991878 |
| RBM47 | 10030.47 | 311.4497 | MAL2 | 17067.62 | 3.967512 |
| ZNF704 | 7044.213 | 215.2375 | MUC16 | 30352.52 | 6.943146 |
| TJP2 | 7307.35 | 221.1888 | KLK6 | 12892.71 | 1.983756 |
| DHCR24 | 42270.32 | 1080.155 | ANO1 | 7374.899 | 0.991878 |
